# Family wellbeing in general practice: a study protocol for a cluster-randomised trial of the web-based resilience programme on early child development

**DOI:** 10.1186/s13063-022-07045-7

**Published:** 2023-01-04

**Authors:** Gritt Overbeck, Jakob Kragstrup, Mette Gørtz, Ida Scheel Rasmussen, Anette Hauskov Graungaard, Volkert Siersma, Sarah de Voss, Ruth Kirk Ertmann, Sinead Shahrzad, Clara Lundmark Appel, Philip Wilson

**Affiliations:** 1 University of Copenhagen, Department of Public Health, Centre for General Practice, University of Copenhagen, Copenhagen, Denmark; 2 Department of Economics and Center for Economic Behavior and Inequality (CEBI), University of Copenhagen, Copenhagen, Denmark; 3 Institute of Applied Health Sciences, University of Aberdeen, Aberdeen, Scotland

**Keywords:** Child development, Internet-based intervention, General practice, Psychosocial factors, Mentalisation

## Abstract

**Background:**

Social, emotional and behavioural problems in early childhood are associated with increased risk for a wide range of poor outcomes associated with substantial cost and impact on society as a whole. Some of these problems are rooted in the early mother-infant relationship and might be prevented. In Denmark, primary health care has a central role in preventive care during pregnancy and the first years of the child’s life and general practice provides opportunities to promote a healthy mother-infant relationship in early parenthood.

**Objective:**

In the context of standardised antenatal and child development assessments focused on psychosocial wellbeing, we examine the impact of a complex intervention designed to improve maternal mentalisation skills, involving training of general practice clinicians and signposting towards a web-based resource. Joint main outcomes are child socio-emotional and language development at age 30 months measured by parentally reported questionnaires (Communicative Development Inventory and Strengths and Difficulties Questionnaire).

**Methods:**

The study is a cluster-randomised controlled trial based in general practices in the Capital Region and the Zealand Region of Denmark. Seventy practices were included. Practices were randomised by a computer algorithm in a ratio of 1:1 to intervention or control groups. Each practice was asked to recruit up to 30 women consecutively at their first scheduled antenatal assessment. Clinicians in both groups received one day of training in preventive antenatal and child development consultations with added focus on parental psychosocial well-being, social support, and parent–child interaction. These preventive consultations delivered in both trial arms require enhanced data recording about psychosocial factors. In intervention clinics, clinicians were asked to signpost a web page at three scheduled antenatal consultations and at four scheduled consultations when the child is 5 weeks, 5 months, 1 and 2 years.

**Discussion:**

We hypothesise that the intervention will increase mothers’ ability to be sensitive to their child’s mental state to an extent that improves the child’s language and mental state at 30 months of age measured by parent-reported questionnaires.

**Trial registration:**

ClinicalTrials.gov NCT04129359. Registered on Oct 16 2019.

**Supplementary Information:**

The online version contains supplementary material available at 10.1186/s13063-022-07045-7.

## Introduction

Studies in birth cohorts with long-lasting follow-up have identified factors associated with poor mental health later in life. These may be genetic, such as vulnerability to ADHD or autism; they may be antenatal (e.g. maternal stress hormones, smoking, and alcohol consumption); they may be located in the family or upbringing, (e.g. postnatal depression, harsh or inconsistent parenting, parental discord); or they may be located in the wider environment (e.g. relative poverty, neighbourhood problems) [1, 2].

These factors may interact in different ways. Some might increase resilience to adversity: in particular, there is a likely protective effect of positive parent-infant interaction against childhood psychological problems [3–7]. Secure infant-parent attachment, itself associated with resilience [8, 9] may be a mediating factor. Early childhood social, emotional and behavioural problems are associated with increased risk of a wide range of poor outcomes associated with substantial cost and impact on society as a whole [10–16]. The association of adverse childhood experiences with long-term ill health is incontrovertible [17, 18].

Childhood language, social and behavioural development predict long-term health [15, 16] and there is a marked overlap between disorders of language development and psychopathology [19–22]. Recent work suggests a stable association between behavioural problems and pragmatic language impairments throughout childhood [23]. It is thus essential to consider language and social, emotional and behavioural difficulties together. Other early markers of general neurodevelopmental vulnerability include abnormalities of motor development [24], sleep disorders, seizures and attention difficulties [25]; conditions which should trigger assessment across the neurodevelopmental domains and lead to careful follow-up.

Parental emotional well-being is another major determinant of a child’s social and emotional development [26, 27]. Cohort research [3–7, 28, 29] demonstrates strong associations between parental mental health, parenting behaviours and children’s psychiatric outcomes. The antenatal maternal mental state may be an even stronger predictor of sensitive parenting behaviours than the postnatal maternal mental state [30]. The mediators of the association between antenatal maternal stress and adverse child outcomes are complex but may involve endocrine effects [31, 32] as well as reduced ‘maternal preoccupation’ with the foetus during a critical period for the development of maternal sensitivity in late pregnancy [30].

The association between postnatal depression and child psychopathology has been long established [33], but the relationship between poor parent–child interaction and poor neurodevelopmental outcomes is probably stronger [34], and treatment of depression alone may be inadequate to achieve improvement in child outcomes [35]. Interventions designed to improve both parental mental health and the parent–child relationship are thus likely to optimise benefits in terms of child development and are potentially valuable public health interventions [36, 37].

Scheduled antenatal and child development assessments offer an opportunity for clinicians to identify potential risks to child neurodevelopment and take appropriate action. These assessments are carried out in diverse settings and by different health professionals internationally [38] but in Denmark, they are largely based in general practice where 10 preventive contacts are offered to families before a child reaches 5 years of age, with high uptake. W, therefore,e decided to test the effectiveness of a general-practice-based intervention designed to improve the child’s psychosocial environment. The intervention is a web-based programme (robustbarn.dk), introduced during practice-based developmental assessments with a psychosocial focus. The programme, signposted to parents when considered appropriate by clinicians, aims to improve parental mentalization skills. Better mentalization skills should help parents to increase their understanding of their own mental state and that of their children, thus improving parent–child interaction and subsequently child developmental outcomes [39].

This protocol paper describes the background, purpose, and design of an effectiveness trial of a complex intervention involving signposting by primary care clinicians towards resources at the robustbarn.dk website during seven scheduled preventive consultations during pregnancy and a child’s first 30 months of life.

### Trial design

This is a cluster-randomised, non-blinded, parallel-group superiority trial with a 1:1 allocation ratio. Enhanced care-as-usual (i.e. including preventive consultations with a structured collection of data on family psychosocial factors) is used as a comparator as this constitutes the most naturalistic approach. A process evaluation and a health economic evaluation will be undertaken during the study period.

## Methods/design

The study is a cluster randomised controlled trial with the general practice as the unit of randomisation. The SPIRIT reporting guidelines were used for this study [40].

### Trial setting

The study is conducted in two of the five Danish administrative regions: the Capital Region and Region Zealand. In Denmark, the healthcare system is free of charge for everyone with a social security number. General practitioners (GPs) are self-employed and work under a collective agreement with the administrative regions. General practices can be singlehanded or consist of several physicians. The GP employs staff, such as practice nurses, midwives, GP trainees, and medical students to deliver services to patients. The GP holds responsibility for all scheduled assessments but can delegate the work to, e.g. a nurse or midwife. The GP functions as a gatekeeper to the secondary healthcare system and offers continuity in preventive childcare with three scheduled antenatal assessments and seven scheduled assessments from birth to school entry. Most communication between GPs and other health services is done through pregnancy charts, referrals, and discharge summaries.

### Intervention and enhanced care-as-usual

**Fig. 1:**
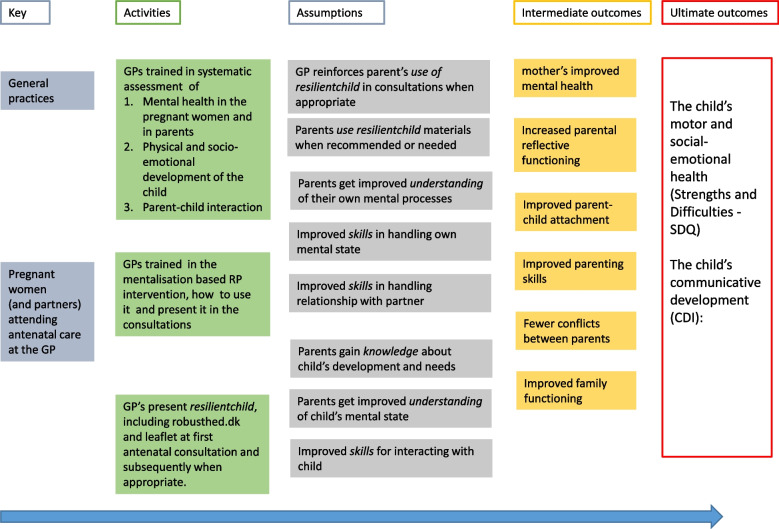
Logic model

The MRC guideline for developing and evaluating complex interventions was used to inform the trial design [41]. (1) We identified existing literature about psychological resilience and the early mother-infant relationship. (2) A programme theory describing the overall rationale for how positive mother-infant relationships could be promoted in the context of scheduled appointments was developed and visualised through the logic model below: (Fig. 1)

- (3) We performed a pilot study between 2017 and 2018. Ten general practitioners participated in a 2-day training course where key concepts from Robusthedsprogrammet (Eng: the resilience programme) [42] were introduced. Participating clinics took part in a discussion that served to refine the resilience programme to match the context of the antenatal assessments in general practice [41]. Lessons learned from the feasibility study led to adjustments of the intervention including reduction of the duration of the training programme for GPs to 1 day; invitation of all clinical staff involved in the assessments to the trial (not only the GP); the introduction of the intervention should fit the context of the first antenatal assessment (at 6–10 week of gestation). This assessment is already burdened with administrative tasks, such as journal recording and choice of birth place, so the initial introduction of robustbarn.dk was limited to 15 min at the first antenatal appointment.

### Identification, eligibility assessment, and recruitment

#### In- and exclusion criteria for GPs

GPs were eligible for participation if they had a clinic registration number in the Capital Region or Region Zealand. GPs that participated in similar trials at the time of inclusion were not eligible.

#### Identification and recruitment of GPs

A list of addresses of every GP clinic in the two Danish administrative regions was retrieved from medcom.dk^1^ in March 2019, and letters were sent to all GP clinics in Region Zealand and the Capital Region inviting them to participate in the study in April 2019. An invitation was also sent as part of an online newsletter to all GPs in the two regions. Clinics received a reminder by email after four weeks. Between May and September 2019, 70 general practices accepted the invitation to participate. They and/or their staff involved in preventive consultations agreed to attend a 1-day or 2-day training program, for control and intervention clinics respectively. All GPs participating in the study received reimbursement for administrative tasks and time spent on courses in connection with the project (standard tariff as negotiated between the GP trade union and the administrative regions).

#### Randomisation of GPs

After completion of GP recruitment but before the training course, GP clinics were randomised to the intervention or the control group. Randomisation was performed by an external statistician using a computer-generated randomisation sequence (evt indsætte navnet på programmet).

##### Inclusion and exclusion criteria for pregnant women

Women were eligible for participation if they were pregnant, ≥ 18 years, and attended their first antenatal assessment in participating general practices. Women were excluded if they are unable to complete questionnaires or participate in the intervention because of very limited Danish language comprehension or if they plan to move to another general practice during the pregnancy or shortly after the birth of the child. Families with other significant difficulties, including those engaged in other therapeutic interventions, were eligible for inclusion.

##### Identification and recruitment of pregnant women

GPs participating in the trial consecutively invited all pregnant women attending their first antenatal assessment, usually in gestation weeks 6–10. Each practice was asked to recruit a minimum of 10 and a maximum of 30 consecutive participants at their first pregnancy assessment starting October 2019. Data were recorded for women who declined participation, and participation rates were monitored carefully.

#### Intervention group

##### 1-day robustbarn.dk-training course for GPs and staff

On the basis of the feasibility study results, a one-day training programme in robustbarn.dk was developed. The training programme was offered to all participating clinics randomised to the intervention group. GPs were encouraged to invite clinic staff usually involved in antenatal assessments and child development assessments to the training course. The course was mandatory for the GP, but voluntary for staff and trainees. It involved introducing the pregnant women to the core concepts of robustbarn.dk as well as encouraging women to log in to the web regularly during pregnancy and after giving birth. The training was provided by specialists in the resilience programme, employed at a government-funded health-promoting organisation “Committee for Health Education” and by a GP with specialist training (AHG) who bridged the use of the intervention elements to fit a general practice setting.

##### Robustbarn.dk

GPs and staff participating in the intervention arm were introduced to the background, the structure and the aim of the website *robustbarn.dk.* Furthermore, they were trained in presenting the intervention to the parents at each preventive examination and in other consultations where the GP considered it likely that the programme could be useful to the family e.g. when they reported mental difficulties during pregnancy or postnatally. All pregnant women in the intervention arm were introduced to the webpage by their GP at the first antenatal appointment. Women also received a leaflet, with a brief description of the website content. Once the GP included a pregnant woman into the project, the woman received a unique login to robustbarn.dk in her safe electronic mailbox (Eboks). This procedure ensured that only women in the intervention group could access the website and thereby should prevent contamination across study arms.

Robustbarn.dk is a website specifically designed for pregnant women and new parents. The content is based on the module-based generic training programme “The resilience programme” [Danish: robusthedsprogrammet] [42].The programme is based on information about mentalisation, cognitive skills, infant and child neurological development and the function of the nervous system, especially during stress or adversity.

The robustbarn.dk website is a collection of brief psycho-educational texts, sound-files, and exercises (please see supplementary material for more information). The intervention includes e-learning modules to parents related to the timing of antenatal and postnatal consultations e.g. information about normal emotional reactions in pregnancy, preparing for delivery, support in relating to the newborn child etc.

##### 1-day assessment-training course for GPs and staff

GPs and staff in the intervention arm additionally received a 1-day training course in the appropriate use of the assessment tools screening for symptoms of depression and anxiety [43], the parent-infant interaction assessment tool [3], infant neuro-developmental assessment [44], child examination and the systematic child record [45].

#### Control group

Pregnant women attended by a GP allocated to the control arm received enhanced care as usual. Control group GPs attended the same 1-day assessment-training course for GP and staff as described above, and were paid to add 15 min to their preventive consultation times to accommodate the extra work. The control group had no insight or training related to the webpage, robustbarn.dk and their patients would not be able to access the website. See Table 1 for an overview of what constitutes the intervention and control groups.

**Table 1:** List of elements constituting the intervention and control groups

***Intervention clinics***	***Control clinics***
• Training in assessing parental mental health, mother-infant-interaction, infant neuro-developmental assessment, systematic child record for all appointments until the child is 2 years	**•** Training in assessing parental mental health, mother-infant-interaction, infant neuro-developmental assessment, systematic child record for all appointments until the child is 2 years
• Instructed in including min. 10 and max. 30 pregnant women consecutively at 1st antenatal appointment	**•** Instructed in including min. 10 and max. 30 pregnant women consecutively at 1st antenatal appointment
• Quarterly newsletters to enhance adherence to the trial	**•** Quarterly newsletters to enhance adherence to the trial
• Training in introducing the concepts of the mentalisation-based robustbarn.dk website	

#### Ensuring adherence

To improve adherence to the protocol, quarterly emails are sent to all participating GPs by the research team enquiring about any problems encountered concerning trial-related tasks. The emails include information on numbers of recruited participants and solutions to potential problems connected to data registration. GPs were paid 1000 Danish Kroner per recruited patient for the clinical time used in the 3-year study period. To secure adequate participant enrolment we asses inclusion every month. All participating GPs receive reminders about the project with regular intervals and those with low inclusion numbers were asked if they needed help with practical issues. To ensure representative enrolment all GPs are asked to make notes about pregnant women who were eligible, but not included. The pregnancy consultation used for inclusion has a special billing code and at the end of the study, it will therefore be possible to perform a register-based analysis of non-participation.

To ensure adherence among the participating pregnant women, three-monthly newsletter emails were distributed during the first 1 year of the project. The newsletter provides updates from the research team and access to an official project website (familietrivsel.dk).

## Outcomes

### Primary outcome

The joint primary outcomes are parentally-reported child social and emotional functioning measured by the Strengths and Difficulties Questionnaire (SDQ) and expressive language performance measured by the MacArthur-Bates Communicative Development Inventory (CDI):

- *Social and emotional functioning* will be measured by the Total Difficulties Scale of the maternally-reported SDQ [46] at age 30 months [19]. The predictive validity for psychiatric disorders 1–2 years later is good, with the area under the Receiver-Operating Characteristic curve (ROC AUC) 0.821 [47]. The SDQ has proved susceptible to change and has been used as a principal outcome in several recent randomised trials reporting successful psychoeducational interventions [48–51]. It is also of note that there are marked differences in SDQ scores at 30 months by socio-economic status: in Glasgow, maternally-reported SDQ Total Difficulties Scale scores are approximately two points higher in the most deprived quintile compared with the least deprived quintile [52].
- *Expressive language performance* will be measured by (CDI) 100-word Danish version [53–59]. This parent-completion questionnaire assesses expressive language performance using a word list [58, 59]. There is a substantial overlap between language delay and psychopathology [14, 19]. Good predictive validity has been reported at 30 months for both language disorder and global cognitive impairment [47] using a similar 50-word list in the UK.

### Secondary outcomes/explanatory variables collected at age 15 months

- Demographic measures
- Accounts of the experience of interventions [explanatory variable]
- Exposure to the intervention (intervention and control groups) [explanatory variable]
- Recent Life Events (RLEQ) [explanatory variable]
- Quality of Life (EQ-5D)
- Maternal anxiety and depression - Hospital Anxiety and Depression Scale (HADS) [secondary outcome]
- Early Symptomatic Syndromes Eliciting Neurodevelopmental Clinical Examinations Questionnaire (ESSENCE-Q) [explanatory variable]

### Secondary outcomes/explanatory variables at age 30 months

- Maternal anxiety and depression - Hospital Anxiety and Depression Scale (HADS) [secondary outcome]
- Child social and emotional development: subscale scores from the Strengths and Difficulties Questionnaire (SDQ) reported by the mother [secondary outcome]
- Maternal health-related quality of life (EQ-5D-5L) [secondary outcome]
- Parental reflective functioning (PRFQ) [explanatory variable]
- Parenting stress (PSI) [explanatory variable]
- Experience of Close Relationships Questionnaire (ECR) [explanatory variable]
- Experience of support (FSS) [explanatory variable]
- Family functioning (FAD) [explanatory variable]
- RLEQ [explanatory variable]

### Long-term outcomes

Data on use of health services, diagnoses and educational attainment will be obtained from national registers.

### Sample size

A total sample of children was estimated to be needed to find a difference of two points in the SDQ Total Difficulties Scale score (an effect size of 0.3) with 80% power at a 2.5% significance level. The estimate of 488 was based on the assumption of an intra-class correlation coefficient (ICC) of 0.02 in 60 clinics (an average of eight children per clinic). Allowing for 22% attrition we therefore aimed to recruit 488/0.78 = 624 children in 60 clusters (of on average 11 children per GP). The ICC estimate was based on the distribution of HADS scores at baseline, suggesting that the impact of clustering effect by practice was modest.

#### Allocation, sequence generation, and concealment

The study is a cluster randomised controlled trial with the general practice as the unit of randomisation. General practices were randomised on a 1:1 basis to intervention or control groups using a computer algorithm. The computer randomised allocation sequence was concealed until all general practices were assigned.

#### Blinding (masking)

The design is open label with only the study statistician being blinded under the dataset locked after collection of primary outcome data so un-blinding will not occur.

### Data collection, management, and analysis

Table 2 shows the SPIRIT timeline of the study.

**Table 2:** The SPIRIT timeline of the study

	**Study period**			
	**Enrolment and allocation**	**Post-allocation pr. pregnant women and newborn baby**		**Close-out**
**Timepoint**	* − 6 months*	*0–14 months*	*15–29 months*	*30–56 months*
**Enrolment of GP clinics**				
**Recruitment general practices**	X			
**Allocation**	X			
**Interventions for the GP:**				
**1 day course**	X			
**Handouts for GPs to give to the women at antenatal and postnatal contacts**	X			
**Individual participants**				
**Pregnant women and subsequently their newborns included in study,**	X	X		
**Assessments:**				
**ACEs** (Adverse Childhood Experiences)	X			
**CSRQ** (Copenhagen Social Relations Questionnaire)	X			
**ECR-Short Form** (Experience in Close Relationship Scale)	X			
**P-PRFQ** (Prenatal Parental Reflective Functioning Scale)	X			
**Demographic measures** (Questionnaire, Questionnaire 2)			X	X
**EQ-5D-5L** (EuroQol, Quality of Life)	X	X	X	X
**HADS** (Hospital Anxiety and Depression Score)	X	X	X	X
**RLEQ** (Recent Life Events Questionnaire)	X	X	X	X
**Use of robustbarn.dk**	X	X	X	X
**ESSENCE-Q-DANISH** (Early Symptomatic Syndromes Eliciting Neurodevelopmental Clinical Examinations Questionnaire)		X	X	X
**FAD** (The McMaster Family Assessment Device)		X	X	X
**FSS** (Family Support Scale)		X	X	X
**PRFQ** (Parental Reflective Functioning Questionnaire)		X	X	X
**CDI-Short Form** (Communicative Development Inventory at 30 months)				X
**SDQ-Dan** (Strength and Difficulties Questionnaire Danish)				X

### Data collection during the study period

The intervention period for each woman is approximately 37 months (~ 7 months pregnancy and the first 30 months of the child’s life). GPs recruited women consecutively and therefore, the 37 months intervention period did not start simultaneously for all included patients. Maternally-reported data (Table 2) are regularly collected from inclusion to the end of the study. Thus, most participant data will be collected at three time-points:

1. Inclusion (baseline)
2. When the child is 15 months and
3. When the child is 30 months

The baseline demographic questionnaire included educational qualification level, employment status, and household composition. All data collected from the women are collected through E-boks, a private and secured online digital mailbox that all citizens in Denmark have.^2^ Baseline data for the study will consist of GP-reported data, patient-reported data, and Danish administrative register data. GP-reported data (including the developmental assessment data) and the families’ self-assessments are completed electronically by use of REDCap [60]. Information about services from the social and health care system will be collected through Danish registers.

#### End-of-study data collection

After completion of the approximately 37 months intervention period for each woman, a questionnaire with primary outcomes (CDI and SDQ) will be sent to the women, followed by the remaining questionnaires. Participants will receive 2 automatic reminders and subsequently a text message to ensure the completeness in data. Mothers’ and their children’s service use will be collected from registers. All data are stored in REDCap [60].

#### Statistical methods

Binary valued outcomes will be analysed in logistic regression and continuously valued outcomes will be analysed in linear regression. To account for clustering within practices and for possible repeated measurements, generalised estimating equations (GEE) will be employed to adjust the covariance matrix. Possible differential dropout will be adjusted for using inverse probability weighting [61].

## Process evaluation

The main objectives of the process evaluation are:

- ○  To identify the key enablers and barriers for signposting the robustbarn.dk programme in clinical practice
- ○  To provide an empirically grounded explanation of the results from the family resilience cluster randomised clinical trial.
- ○  To contribute to the family resilience project’s overall assessment of the value, implications, and potential scalability and transferability of the intervention.

These objectives involve monitoring and exploring how professionals and patients respond to the intervention (with what type of consequences), how the intervention is actually implemented in practice (if it is implemented as intended in the protocol, i.e. implementation fidelity), and how contextual factors influence implementation processes, mechanisms, and outcomes [62].

The implementation process will be evaluated using Normalization Process Theory (NPT), which provides an explanatory framework for investigating how complex interventions are implemented in organisational settings. According to the theory, implementation emerges through four generative mechanisms: coherence, cognitive participation, collective action, and reflexive monitoring [63, 64].

### Process evaluation data collection and analysis

The study will adopt a mixed-methods approach combining in-depth qualitative data with quantitative process data on intervention activities [62].

#### Qualitative interviews and observations

During and after the randomised clinical trial, semi-structured, face-to-face interviews will be conducted with purposively sampled health professionals and patients from 15 practices. The aim is to conduct approximately 20 interviews with health professionals and 20 interviews with their patients. The interviews will be audio-taped and transcribed verbatim. First, the data material will be analysed using an inductive thematic approach, and subsequently, a more deductive thematic analysis will be performed using NPT as a coding framework.

#### Quantitative data

This qualitative study will be supplemented by descriptive quantitative data on key process indicators, preferably from all sites or participants, for example number and duration of interventions website visits. Women’s use of the homepage during the first year after entering the study will be analysed in order to describe how their characteristics are associated with the use of the intervention. Further, data about the general practices will be assessed to examine how different practice characteristics affect the women’s use of the intervention. The following data regarding general practice characteristics will be handled; practice area deprivation, practice organisation categorised as single-handed practice, companionship practice or group practice, practices with or without a nurse or midwife and how many women each practice had recruited.

#### Interpretation of qualitative and observational data

Qualitative data will be coded according to the framework of Normalization Process Theory and will address the main objectives described above [65].

## Economic evaluation

The economic cost analysis will consider and present possible costs and benefits associated with the intervention. In particular, the analysis will estimate the costs of the treatment as compared with the costs of the standard care offered to the control group. Furthermore, the economic analysis will assess the benefits of the treatment vis-à-vis standard treatment, e.g. in terms of potential saved short- and long-term costs. To the extent possible, the economic analysis will thus examine the long-term benefits and costs of the intervention.

Costs and benefits will be assessed using linked information from survey respondents to socio-economic and health information in the Danish registers. Linking to register data will facilitates following individuals (parents and children) over time, and the survey will thus be an invaluable source for future follow-up research. The analysis will take a societal perspective to include costs that fall on GPs, other relevant service providers (for example, health visitors or social services) and the affected mothers and their families. Costs of the intervention will be obtained from trial documentation and in consultation with intervention providers. The intervention costs will include GP cost for delivering any relevant components of the intervention, RP staff costs for delivering training, and costs of any consumables required to deliver interventions. Costs of usual care include costs of current care to affected mothers provided by health visitors (sundhedsplejerske) and general practitioners, and also the cost falling on other services (hospital, local authority or other services) through referrals. These data will be combined with study-specific unit costs or unit costs from publicly available standard sources to produce a total cost for both the intervention and control groups.

Apart from changes in the child and maternal outcomes, there are likely to be wider benefits. These might include work-related or educational benefits for the affected mothers and their families, increased family cohesion, potential reduction of inequalities between socioeconomic groups, as well as better educational outcomes (eventually) for the children involved. Those benefits may come about as a result of increasing interactions between mothers and their children and other family members, and increased knowledge and skills for continued improved functioning in the future. We shall also collect EQ-5D-5L data from mothers at baseline, every 6 months, and at the end of the trial to capture potential improvement in maternal quality of life.

A sensitivity analysis will be undertaken to explore possible variations of outcome measures and estimate mean effects as well as confidence bands.

As suggested, it is possible that the intervention may lead to long-term benefits to society beyond the trial’s follow-up period. A longer time horizon will provide more time for the effects to accrue and potentially offset the initial costs of the intervention. The long-term benefits of the intervention may include costs saved as a result of conduct and emotional disorders avoided, avoided criminal justice costs, reduced needs for special educational services, reduced mental health service use, and reduced productivity loss for the family as well as improved quality of life for parents. Using longitudinal register data allows for such long-term follow-up.

## Data management

The study adheres to all Danish laws governing medical research. The General Data Protection Regulation is upheld, and data are stored and handled accordingly. The study owner (University of Copenhagen) is responsible for upholding laws and ensuring the confidentiality of data. All data that can identify participants is encrypted and stored securely on password-protected servers with continuous transaction logging. Trial data are stored in accordance with the data policy of the University of Copenhagen. Data are saved for 5 years after data collection and will thereafter be anonymized or deleted.

### Monitoring

The study has been approved by the University of Copenhagen Data Protection Agency (Case no. 514–0362/19–3000). According to Danish legislation, there is no need to apply for the approval of the National Danish Data Protection Agency when regional approval has been given. A data processing agreement with each GP has been signed before the collection of data. The project management group have met once a month throughout the trial period to monitor recruitment, trial progress, completeness of data and ethical issues. Our Trial steering committee met at the outset of the trial and made a decision to meet on an ad-hoc basis when requested by the investigating team. This has not been required to date, but the TSC will be convened when our final dataset is locked.

## Ethics and dissemination

### Research ethics approval

The trial has been approved by the University of Copenhagen Research Ethics committee for Science and Health (ref. 504–0111/195000). This trial does not involve collecting biological specimens for storage.

### Protocol amendments

The trial will be conducted in accordance with the protocol. If modification is required, the funder will be notified, followed by the clinics involved in the project. Significant changes will be registered here: https://clinicaltrials.gov/ct2/show/NCT04129359. Minor changes, seen as administrative rectifications to the protocol with no effect on the conduct of the trial will be agreed upon by the project management group.

### Dissemination policy

The results from the study will be published in peer-reviewed journals. The final list and order of authors follow the contribution from each researcher and follows the Vancouver rules and the guidelines from The Danish Committees on Scientific Dishonesty. PW is the Chief Investigator; he conceived the study, led the proposal and protocol development together with JK. GO, MG, AG and VS, contributed to the study design and to the development of the proposal. PW, VS, GO, AG and JK and VS were the lead trial methodologists. All authors read and approved the final manuscript.

### Access to data

After our primary publication of the trial results, a version of the data will be shared on a public platform and made available for research in accordance with Danish law about the protection of personal data.

## Discussion

This cluster-randomised controlled study aims to test the effectiveness and feasibility of an intervention to increase mental well-being and resilience in new mothers and their offspring. GPs and staff received brief training in the core concepts of the web-based resilience training programme. From October 2019 to March 2020 70 GPs/staff were trained and subsequently included pregnant women in the study. An obvious shortcoming of the study might be related to COVID-19, since a large proportion of antenatal and postnatal contacts were reported to be affected by the pandemic. GPs and staff reported that pregnant women’s major concerns were infection and infection-related risks. This delimited the clinicians’ window of opportunity to put mental well-being and mentalisation on the agenda during consultations, and the robustbarn.dk intervention might have been introduced with less enthusiasm than might have occurred otherwise due to other competing tasks in the clinic.

### Strengths

The location of this pragmatic cluster-randomised trial in Danish general practice, with its typically high levels of engagement with preventive obstetric and child developmental assessments, is likely to create a robust sample with relatively low levels of attrition assuming patients remain registered with their original GP. Recruitment of consecutive patients attending their first antenatal appointment should provide a sample representing a typical clinical caseload for participating practices. The flexibility given to clinicians in terms of signposting their patients towards the web-based resources should reflect potential future real-world practice underpinned by progressive universalist principles [66] and the quantitative process evaluation of website usage will allow assessment of equity of access to the resources. The strength of Danish population registers will ensure reliable long-term follow-up data for almost all participants.

### Limitations

It was necessary to exclude non-Danish-speaking women from participation for pragmatic reasons, and this will reduce the generalizability of findings to migrant populations.

It is possible that there may be variation in recruitment rates across practices — with some practices generating selective samples, while others may recruit almost all eligible women [67]. Similarly, there may be variation across practices in the extent to which the intervention is presented to participants. Given the design of the trial, these factors may increase clustering effects and potentially reduce statistical power.

There may be some dilution of the intensity of intervention if participants change practice or consult with untrained clinicians within their existing practice.

## Trial status

Protocol version 2, 23.09.2020.

Initiation of recruitment of patients by October 2019. Expected to be completed before the end of 2022.

The SPIRIT checklist and timeline have been included as additional information and in the text.

## Supplementary Information

**Additional file 1. **Web content description.

**Additional file 2. **Trial set data registration.

## Data Availability

The research team will have full access to all data.

## Abbreviation

GP: General practitioners

## Footnotes

1
https://www.medcom.dk/medcom-in-english

2
https://www.e-boks.com/danmark/en/what-is-e-boks/
