# Supplementary material for: Family wellbeing in general practice: a study protocol for a cluster-randomised trial of the web-based resilience programme on early child development
Source: Trials. 2023 Jan 4;24:7. doi: 10.1186/s13063-022-07045-7 (PMC9810520; doi:10.1186/s13063-022-07045-7)
Supplement: Supplementary file 1 — Additional file 1. Web content description. [file 13063_2022_7045_MOESM1_ESM.pdf]

Robustbarn.dk – core content

The program consists of **seven modules** with written information, examples, sound files and exercises.

Each module can be accessed through five main entrances: “Pregnancy”, “Birth”, “Child 1 year”, “Child 2 years” and “Farther/partner”. This makes it easier for the parents and the GPs to reach the content relevant for specific situations.

Each of the five contain a short description of how this program may support the user at this specific period of his/her life; a short description of the conditions and challenges that are common at this stage regarding e.g. bodily experiences, social relations or roles. Then there is a short presentation of evidence-based knowledge about the psychological and relational processes in this period is presented. Finally frequent challenges are addressed e.g. pregnancy complications or difficulties in relating to the child etc. In each section there are links into the relevant content, exercises or sound files

The modules contain the following topics: “Mentalisation”, “The brain”, “Thoughts”, “The body”, “Awareness”, “Resilient relations”, “Challenges”.

1. The **Mentalisation** module describes briefly the concept of mentalisation, a short mentalisation exercise and a systematic description of the development in the child from 0-4 years.
2. The **Brain module** contains a simple description of the alarm system in the brain and the cooperation between the alarm system and the cognitive system with examples and figures. The role of the oxytocin hormone in calming the nervous system is described and the development of the brain in the child 0-2 years is shortly presented with a focus on the alarm system.
3. The **Thoughts module** highlights the fact that thoughts are nothing else but thoughts and thus possible to notice, categorise and maybe change. There are several exercises and figures to illustrate this.
4. In **The Body module** the relationship between the body and the mind are repeated with specific focus on alarm mood states. There are breathing exercises and suggestions for self-care.
5. In **the Awareness module,** awareness is described as the spotlight we all use to focus the mind on something specifically and how it is possible to move the awareness to something else by deciding it or training the mind to move from one focus to another.
6. The **Resilient relations module** set focus on the strengths and importance of social relations in the challenging transition into parenthood and presents a short dyadic exercise to support the relation.
7. The **Challenges module** address that we all need challenges that makes us grow but not so difficult that we get into an alarm situation where we cannot solve any problems, e.g. as a new parent with a screaming baby. The importance of the parents presenting the child with age specific challenges to develop autonomy in the child is described. Specific techniques to support and acknowledge the child’s attempts of development are suggested
