# Supplementary material for: Family wellbeing in general practice: a study protocol for a cluster-randomised trial of the web-based resilience programme on early child development
Source: Trials. 2023 Jan 4;24:7. doi: 10.1186/s13063-022-07045-7 (PMC9810520; doi:10.1186/s13063-022-07045-7)
Supplement: Supplementary file 2 — Additional file 2. Trial set data registration. [file 13063_2022_7045_MOESM2_ESM.docx]

**Table 1:** **Data set, trial registration**

| Data category | Information |
| --- | --- |
| Primary registry and trial identifying number | ClinicalTrials.gov: NCT04129359 |
| Date of registration in primary registry | October 16, 2019 |
| Secondary identifying numbers | TRYG: 125227  KEU: 19035774 |
| Source(s) of monetary or material support | TRYGFoundation (ah@trygfonden.dk)  The Quality and Educational Committee (KEU; Kvalitet og Efterudannelsesudvalget) Capital Region of Denmark (kap-h@regionh.dk) |
| Primary sponsor | TRYGFoundation |
| Secondary sponsor(s) | The Quality and Educational Committee (KEU; Kvalitet og Efterudannelsesudvalget) Capital Region of Denmark |
| Contact for public queries | Gritt Overbeck grio@sund.ku.dk |
| Contact for scientific queries | Gritt Overbeck grio@sund.ku.dk |
| Public title | FamilieTrivsel i Almen Praksis: a Mentalisation Programme for Families With Young Children |
| Scientific title | A general practice-based cluster-randomised trial of the impact of the Resilience Programme on early child development |
| Countries of recruitment | Denmark |
| Health condition(s) or problem(s) studied | Mental health, child development |
| Intervention(s) | Usual care plus a version of the Robusthed.dk mentalisation-based web resource, recommended by intervention group GPs compared with usual care alone. |
|  | Control: enhanced care as usual |
| Key inclusion and exclusion criteria | Ages eligible for study: ≥16-50 Sexes eligible for study: women and their children Accepts healthy volunteers: yes |
|  | Inclusion criteria: All women attending for their first antenatal assessment in participating general practices |
|  | Exclusion criteria: Pregnant women without adequate Danish language skills to complete the assessments or to use the intervention. |
| Study type | Interventional |
|  | Allocation: This trial is designed as a cluster randomised, non-blinded parallel group superiority trial with 1:1 allocation ratio. Enhanced care-as-usual will be used as a comparator. |
|  | Primary purpose: Supportive care |
|  | Study Phase: n/a |
| Date of first enrolment | October 29, 2019 |
| Target sample size | 488 |
| Recruitment status | All participants recruited |
| Primary outcome(s) | Child language (50 word version of the MacArthur Communicative Development Scales) and socio-emotional development (Total Difficulties Scale from the Strengths and Difficulties Questionnaire); |
| Key secondary outcomes | Secondary outcomes include maternal mental health (Hospital Anxiety and Depression Scale); parent-child interaction quality assessed by the Child-Adult Relationship Observation. |
